# Variation in Practice Pattern of Male Hypogonadism: A Comparative Analysis of Primary Care, Urology, Endocrinology, and HIV Specialists

**DOI:** 10.1177/1557988317743152

**Published:** 2017-11-28

**Authors:** Yash S. Khandwala, Omer A. Raheem, Mir Amaan Ali, Tung-Chin Hsieh

**Affiliations:** 1 Department of Urology, University of California San Diego, La Jolla, CA, USA; 2 University of California San Diego School of Medicine, La Jolla, CA, USA

**Keywords:** hypogonadism, physiological and endocrine disorders, low testosterone syndrome, physiological and endocrine disorders

## Abstract

The objective of the current study was to measure the adherence of guideline-based evaluation and treatment of hypogonadism by medical specialty. A retrospective review was performed analyzing patients from a single academic institution within the past 10 years. The cohort of 193 men was grouped according to medical specialty of the diagnosing physician (50 urology, 49 primary care, 44 endocrinology, and 50 HIV medicine). Adherence to guidelines was assessed using the Endocrine Society’s criteria. Primary care patients were older compared to the rest of the cohort (*p* < .001) but BMI and cardiovascular risk factors were similar (*p* = .900). Patients treated by urologists and endocrinologists had the highest percentage of low testosterone findings at initial encounter at 72% (*p* < .001). Sixty-two percent of urology patients had low LH or FSH compared to 63.6% for endocrinology and 16% for primary care (*p* < .001). As for brain MRI findings, no urology patients had positive findings (0/9) while eight pituitary adenomas (40%) were found by endocrinologists. Forty-five percent of men treated by urologists received TRT without repeat confirmation, compared to 58% of endocrinologists, 77% of primary care, and 88% of HIV medicine (*p* < .001). All urology patients had PSA checked before TRT compared to 77.5% of primary care and 61.2% of endocrinology patients (*p* = .063). Adherence to the guidelines helps prevent undue over-diagnosis and over-treatment of hypogonadism. This study suggests that adherence to guideline-based screening is varied among specialties.

With an aging population in the United States, hypogonadism is on the rise. By 2020, 55 million men older than 65 years are expected to be afflicted, with estimates rising to 87 million in 2050 (Seftel, 2006). The advancing prevalence of this disease has led to an increased effort to investigate the pathophysiology, morbidity, and mortality associated with testosterone deficiency. Reduced serum testosterone concentration has been reported to adversely affect male physiology resulting in significantly reduced quality of life. These men may suffer from disruption in anabolic muscle growth, bone strength, mood and cognition, and red blood cell production on a spectrum ranging from mild discomfort to significant debility. Hypogonadism has also been associated with major comorbidities such as cardiovascular events, diabetes, and metabolic syndrome (Dandona & Rosenberg, 2010).

The use of testosterone replacement therapy (TRT) for the management of hypogonadism has evolved drastically since its inception nearly 75 years ago (Heller & Myers, 1944). Recent evidence has confirmed that TRT does indeed improve libido, sexual activity, and erectile function in men with low baseline testosterone levels (Bhasin et al., 2010). Over the past two decades, transdermal, and intramuscular routes of delivery have thus become exceedingly popular (Nieschlag & Nieschlag, 2014). Between 2001 and 2011, the use of exogenous androgens increased more than 300% (Baillargeon, Urban, Ottenbacher, Pierson, & Goodwin, 2015). Over the past 5 years, there has been increasing concern regarding the potential overuse of TRT for off-label indications such as physiological aging and idiopathic loss of libido (Gan, Pattman, Pearce, & Quinton, 2013). In 2014, the US Food and Drug Administration (FDA) advised caution in prescribing testosterone without confirmation of low serum levels and an associated medical condition as there may be an increased risk of myocardial infarction, stroke, and mortality in some patients undergoing TRT (Finkle et al., 2014; Vigen, O’Donnell, Barón, Grunwald, & Maddox, 2013).

Despite growing evidence suggesting the importance of careful management of hypogonadism, there remains a lack of specialty-specific guidelines for testosterone replacement (Aversa & Morgentaler, 2015). The prevailing guidelines currently used by most clinicians were established in 2006 by the Endocrine Society (ES) and last updated in 2010 (Bhasin et al., 2006, 2010). The American Urological Association (AUA) revised their position on testosterone therapy in 2015, but it consists mainly of adaptation from the recommendations published by the ES.

Furthermore, it remains unclear how many physicians prescribing testosterone are indeed following these established guidelines and if these guidelines are applicable to non-endocrine specialties. The objective of this study was to determine interdisciplinary rates of adherence to established recommendations for the diagnosis and management of hypogonadism and the clinical utility of these guidelines for urologists, primary care physicians (PCPs), endocrinologists, and HIV specialists at a single academic institution.

## Methods

### Study Cohort

Using an internal institutional database of patients from a single U.S.-based academic center identified using the International Classification of Diseases, Ninth Revision (ICD-9) coding system, patients presenting for care in 2016 with a diagnosis of hypogonadism (257.2) were randomly selected. These patients were subsequently matched by the managing medical subspecialty resulting in relatively even subgroups. Men with a history of opioid use, testicular cancer, and chronic corticosteroid or anabolic steroid use; incomplete or missing data; and primary evaluations taking place before 2006 were excluded. Men were randomly selected until 50 charts from patients of each specialty were reviewed.

### Determination of Adherence to Guidelines

To assess interdisciplinary adherence to the established guidelines, observed practice patterns of the providers were compared with those recommended by the Endocrine Society’s Clinical Practice Guidelines published in 2006 and updated in 2010 (Bhasin et al., 2006, 2010). The guidelines suggest that before prescribing TRT, providers should twice confirm low morning serum total testosterone levels after excluding reversible illness, drugs, or nutrition deficiency as a cause of low testosterone levels. The repeat testosterone screen should be accompanied by a test for serum luteinizing hormone (LH) and follicle stimulating hormone (FSH). Free or bioavailable testosterone can also be used to determine low values if sex hormone-binding globulin (SHBG) is thought to be abnormal. If LH or FSH is low or normal—suggesting secondary hypogonadism—then prolactin/other pituitary hormone levels are recommended.

The ES guidelines suggest using 300 ng/dL as the lower cutoff for normal testosterone and 5 ng/dL for free testosterone. LH and FSH are considered abnormally low if less than 5 mlU/mL and prolactin is considered abnormally high if serum concentrations are above 20 ng/mL. Pituitary imaging is indicated if serum testosterone is below 150 ng/dl; panhypopituitarism is noted, or hyperprolactinemia is evident (Bhasin et al., 2006, 2010). These cutoff values were thus used to determine results in the abnormal range for this study. Laboratory values were only included in the analysis if the tests were drawn within 3 months of the initial clinic visit during which the diagnosis of hypogonadism was made. All charts were thoroughly reviewed to ensure that all completed tests were captured.

### Statistical Analysis

**Figure 1.**
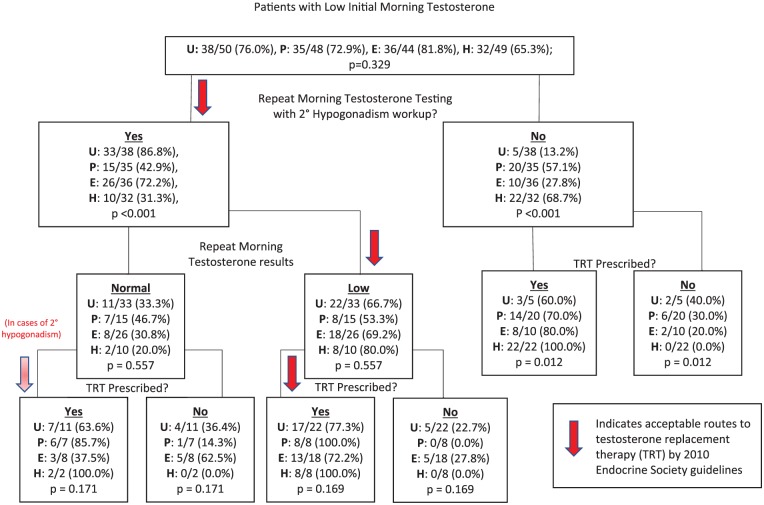
Step-by-step evaluation of adherence to ES guidelines by specialty.

Patient demographics (age and BMI) and relevant comorbidities (diabetes, thromboembolic disease, coronary artery disease (CAD), HIV, erectile dysfunction (ED), and prostate cancer) were extracted and compared. The analysis of variance (ANOVA) test was used for comparison between groups. A flowchart (Figure 1) was constructed to depict each specialty’s pattern of care for patients that were diagnosed with hypogonadism. After each management decision, a Pearson’s χ^2^ test or Fisher’s exact test (if expected values were fewer than five patients) was performed to determine if a difference in management paradigm existed between each of the four specialties (Figure 1). This flowchart was subsequently compared with the established guidelines previously mentioned to determine adherence. Prostate-specific antigen (PSA), hemoglobin (Hgb) concentration, liver function tests, and pituitary hormone levels were compared for patients ultimately receiving TRT. The most common method of testosterone replacement was also compared between specialties.

The percentage of patients receiving LH, FSH, and prolactin testing were compared as well as patients receiving brain MRI for the appropriate indications. A sensitivity analysis on the mean serum pituitary hormone concentrations of patients with pituitary masses was also conducted and means were compared using ANOVA. All statistical analyses were conducted using STATA version 14 (College Station, TX). All tests were two-sided and utilized a significance level of *p* < 0.05.

## Results

A total of 193 patients remained for the current analysis with 50 (25.9%) diagnosed by urologists, 49 (25.4%) by PCPs (internal medicine or family medicine), 44 (22.8%) by endocrinologists, and 50 (25.9%) by HIV specialists. HIV patients were significantly younger than patients diagnosed by urologists and PCPs (45.7 vs. 53.5 and 60.5 years, respectively; *p* < .001). Men diagnosed by endocrinologists had the highest mean BMI (30.4) while HIV patients had the lowest (26.1) (*p* = .003). No significant difference was noted in mean PSA (urology: 1.0 ng/mL, primary care: 1.2 ng/mL, endocrinology: 0.7 ng/mL, HIV medicine: 1.6 ng/mL; *p* = .548) and Hgb (urology: 14.2 g/dL, primary care: 14.3 g/dL, endocrinology: 13.8 g/dL, HIV medicine: 16.5 g/dL; *p* = .437). Patients seen by endocrinology and HIV medicine had higher mean serum aspartate transaminase (AST) and alanine transaminase (ALT) levels compared to patients who presented to urologists or PCPs (AST: 30.6 and 31.2 units/L vs. 23.8 and 24.8 units/L, *p* = .027; ALT: 32.1 and 37.2 units/L vs. 25.2 and 25.9 units/L, *p* = .010, respectively). There were no significant differences noted between the percentage of the four cohorts with diabetes, thromboembolic disease, coronary artery disease, erectile dysfunction, and prostate cancer. All 50 patients (100%) diagnosed with hypogonadism by HIV specialists were HIV positive. As for the other patients, five patients (11.4%) seen by endocrinologists, three (6.0%) seen by urologists, and two (4.0%) by PCPs were positive for HIV (Table 1a and b).

**Table 1a.** Patient Characteristics by Managing Specialty [mean (SE)].

	Urology	Primary care	Endocrinology	HIV medicine	*p*
*N* (%), patients	50 (25.9%)	49 (25.4%)	44 (22.8%)	50 (25.9%)	
Age (years)	53.5 (2.5)	60.5 (2.1)	48 (2.2)	45.7 (1.5)	<.001
BMI (kg/m^2^)	28.8 (0.7)	28.9 (0.8)	30.4 (1.0)	26.1 (0.8)	.003
PSA (ng/mL)	1.02 (0.2)	1.2 (0.2)	0.7 (0.2)	1.6 (0.8)	.548
Hgb (g/dL)	14.2 (0.5)	14.3 (0.3)	13.8 (0.6)	16.5 (2.1)	.437
AST (units/L)	23.8 (1.6)	24.8 (1.5)	30.6 (3.2)	31.2 (1.9)	.027
ALT (units/L)	25.2 (2.9)	25.9 (1.8)	32.1 (3.4)	37.2 (3.3)	.010

*Note.* BMI = body mass index; PSA = prostate-specific antigen; Hgb = hemoglobin; AST = aspartate transaminase; ALT = alanine transaminase.

**Table 1b.** Patient Comorbidities [number (%)].

	Urology	Primary care	Endocrinology	HIV medicine	*p*
Diabetes	9 (18.0)	8 (16.0)	15 (34.1)	15 (30.0)	.115
Thromboembolic disease	10 (20.0)	10 (20.0)	3 (6.8)	6 (12.0)	.190
Coronary artery disease	10 (20.0)	8 (16.0)	3 (6.8)	6 (12.0)	.288
HIV	3 (6.0)	2 (4.0)	5 (11.4)	50 (100)	<.001
Erectile dysfunction	22 (44.0)	21 (42.0)	14 (31.8)	13 (26.0)	.181
Prostate cancer	5 (10.0)	2 (4.0)	1 (2.3)	0 (0.0)	.076

While all patients in the cohort were diagnosed with hypogonadism, not all men had low morning testosterone upon initial testing. Only 35 patients (72.9%) diagnosed by PCPs and 32 patients (65.3%) diagnosed by HIV specialists had initial low testosterone compared to 38 patients (76.0%) diagnosed by urologists and 36 (81.8%) by endocrinologists (*p* = .329). Of patients with low initial testosterone, those diagnosed by urologists and endocrinologists underwent confirmatory testing significantly more often (86.8% and 72.2%, respectively) than men diagnosed by PCPs and HIV specialists (42.9% and 31.3%, respectively) (*p* < .001). No significant difference was noted between specialties in TRT prescribing habits irrespective of if repeat testing resulted in low or normal levels of testosterone. However, urologists were the least likely specialty to prescribe TRT without a second confirmatory low testosterone reading (urology: 45.5%, primary care: 77.5%, endocrinology: 58.1%, HIV medicine: 88.0%; *p* = .012). Urologists prescribed Androgel and testosterone Cypionate formulations most frequently (10/34 patients (29.4%), respectively) while endocrinologists and HIV specialists preferred Cypionate (17/31 (54.8%) and 22/50 (44.0%), respectively); PCPs tended to prescribe Androgel most often (23/39 (59.0%); *p* < .001). Figure 1 compares hypogonadism management for each specialty with established ES guidelines using a flow chart.

Urologists screened for PSA before prescribing TRT in all 33 patients (100%), more often than the 30/39 (76.9%) for PCPs, 19/31 (61.3%) for endocrinologists, and 34/50 (68.0%) for HIV specialists (*p* = .001). All specialties consistently ordered complete blood counts (CBC) and liver function tests (LFTs).

The mean initial and repeat, total and free testosterone concentrations were not significantly different among specialties. However, patients managed by HIV specialists had the highest mean SHBG concentrations (urology: 38.4 nmol/L, primary care: 36.1 nmol/L, endocrinology: 35.3 nmol/L, HIV medicine: 50.9 nmol/L; *p* < .001). Of the patients who were screened for low pituitary hormone concentrations, those diagnosed by urologists and endocrinologists had the lowest mean serum FSH (5.7 and 5.4 mlU/mL, respectively) compared to 14.0 mlU/mL for primary care and 12.9 mlU/mL for HIV specialists (*p* = .038). Patients diagnosed by endocrinologists had significantly higher mean serum prolactin concentrations (221.4 ng/mL) compared to their counterparts (urology: 12.2 ng/mL, primary care: 9.7 ng/mL; *p* < .001). No patients treated by HIV physicians had their prolactin levels measured (Table 2).

**Table 2.** Mean (SE) Baseline Laboratory Values by Specialty of Treating Physician.

	Urology	Primary care	Endocrinology	HIV medicine	*p*
Serum steroid concentrations					
Initial total testosterone (ng/dL)	272.18 (25.7)	257.38 (18.5)	226.68 (25.7)	297.02 (23.7)	.218
Initial free testosterone (ng/dL)	50.00 (6.0)	44.99 (4.7)	44.51 (5.6)	52.35 (4.7)	.696
Repeat total testosterone (ng/dL)	360.10 (46.6)	295.56 (46.0)	260.77 (37.5)	352.26 (58.7)	.368
Repeat free testosterone (ng/dL)	74.03 (13.0)	47.10 (7.0)	39.06 (9.9)	64.42 (5.7)	.221
SHBG (nmol/L)	38.43 (3.4)	36.07 (3.2)	35.27 (4.4)	50.9 (7.6)	<.001
Pituitary hormones concentration					
LH (mlU/mL)	4.40 (.53)	8.95 (3.3)	4.38 (.72)	5.9 (1.9)	.074
FSH (mlU/mL)	5.72 (.86)	13.98 (6.1)	5.38 (1.3)	12.93 (8.5)	.038
Prolactin (ng/mL)	12.23 (2.5)	9.72 (1.46)	221.38 (147.8)	n/a	<.001

*Note.* SHBG = sex hormone-binding globulin; LH = luteinizing hormone; FSH = follicle stimulating hormone.

In general, urologists and endocrinologists performed screening for secondary hypogonadism more consistently (urology: 78.0%, primary care: 26.5%, endocrinology: 75.0%, HIV medicine: 6.0%; *p* < .001). However, endocrinologists ordered brain MRIs on patients diagnosed with hypogonadism more often (urology: 18.0%, primary care: 10.2%, endocrinology: 75.0%, HIV medicine: 6.1%; *p* < .001). While eight patients (40%) from the endocrinology cohort had pituitary abnormalities on imaging, none of the nine patients sent by urologists resulted in positive findings (*p* = .140) (Table 3).

**Table 3.** Number (%) of Patients Receiving Pituitary Screening.

	LH or FSH	Prolactin	Brain MRI	Positive brain MRI
Urology	39 (78.0)	31 (62.0)	9 (18.0)	0 (0.0)
Primary care	13 (26.5)	12 (24.5)	5 (10.2)	1 (20.0)
Endocrinology	33 (75.0)	28 (63.6)	20 (45.5)	8 (40.0)
HIV medicine	3 (6.0)	1 (2.0)	3 (6.1)	1 (33.3)
*p*	< .001	< .001	< .001	.140

*Note.* LH = luteinizing hormone; FSH = follicle stimulating hormone; MRI = magnetic resonance imaging.

## Discussion

Patients diagnosed with hypogonadism by urologists, PCPs, endocrinologists, and HIV specialists all appeared to have similar baseline characteristics. Irrespective of specialty, all providers had poor adherence to established guidelines though urologists least often prescribed testosterone without a repeat testosterone screen and most consistently screened for PSA prior to TRT. Patients diagnosed and treated by HIV specialists tended to have higher mean serum SHBG concentrations while urology and endocrinology patients had lowest levels of FSH. Furthermore, as expected, prolactin was highest in the endocrinology patients with abnormal pituitary MRI findings. Urologists and endocrinologists most frequently screened for secondary hypogonadism and suprasellar abnormalities per established guidelines.

In recent years, there has been considerable global concern for the over prescription of testosterone due to both a lack of universal hypogonadism management guidelines and a failure to adhere to established ones. This concern arises from a knowledge of the risks inherent to TRT (Bassil, Alkaade, & Morley, 2009; Gilbert, Cimmino, Beebe, & Mehta, 2017; Rhoden & Morgentaler, 2004). In Korea, increase in prescriptions by both PCPs and urologists have led to heightened efforts to understand the effects of exogenous testosterone on Asian men with the intention of developing race-specific guidelines (Ko & Kim, 2011). In the UK, due in part to the high uptake of transdermal testosterone preparations, TRT increased 90% between 2001 and 2010 despite the prevalence of hypogonadism remaining constant, suggesting an increase in off-label use (Gan et al., 2013). The risks associated with exogenous testosterone in the United States have been well delineated, but there still appears to be inconsistencies in practice patterns. A large study of Veterans Affairs (VA) patients published in 2015 reported only 3.1% of men receiving testosterone had two or more low morning testosterone levels with LH/FSH testing. 16.5% did not have levels checked at all. And possibly due to a lack of consensus on the association of TRT and prostate cancer risk, PSA was only measured in 76% of patients receiving exogenous hormone (Jasuja, Bhasin, Reisman, Berlowitz, & Rose, 2015). Unfortunately, this latter study failed to characterize which providers or medical specialties were involved in the prescribing of testosterone.

The low rate of adherence to hypogonadism management guidelines (mentioned previously) in this study and in the literature may suggest suboptimal quality of care for some patients. A landmark paper published in the New England Journal of Medicine in 2010 highlighted potential adverse events that patients receiving exogenous testosterone may be at higher risk for such as cardiovascular, respiratory, and dermatologic events (Basaria, Coviello, Travison, & Storer, 2010). Indeed, TRT has also been linked to altering prostate cancer risk and severity, though this relationship is complex and remains unclear (Eisenberg, 2015).

Despite the overall poor adherence to the Endocrine Society guidelines, urologists appeared to be the most judicious prescribers of T while PCPs and HIV specialists deviated from ES recommendations most often. Of the population of urologists sampled it was noted that there were a number of fertility specialists who were particularly adherent to the guidelines and saw proportionally higher volume of patients suffering from hypogonadism. One possible explanation for the perceived overuse of testosterone by HIV providers is the utility of short-term TRT for management of HIV-associated weight loss, wasting, depression, and loss of muscle referenced by the ES guidelines (Bhasin et al., 2006, 2010). HIV patients also tend to have higher levels of SHBG on average which may mask true hypogonadism if free testosterone is not tested, which underpins the importance of guideline adherence (Atan, Tuncel, Yesil, & Balbay, 2013; Martin, Benassayag, Amiel, Canton, & Nunez, 1992). Perhaps the needs of the HIV patient population are not sufficiently addressed by the ES guidelines, and the utility of TRT lends itself especially well to patients who would normally not be recommended TRT by the ES guidelines.

These findings suggest a need for further investigation into the value of specialty or patient population-based guidelines. Physicians may be more adherent to guidelines endorsed by their own respective fields. Furthermore, while all patients undoubtedly benefit from careful and diligent prescription of testosterone, the screening for pituitary tumors may not be equally useful among all populations. Indeed, urology and endocrinology patients had the lowest mean FSH levels while endocrinology patients had significantly higher mean prolactin levels. However, possibly due to a small sample size, there was no statistically significant difference in patients with positive brain MRI findings among specialties. Large, randomized controlled studies are requisite to accurately compare the utility of brain imaging and other expensive screening tools in patients with suspected hypogonadism.

There are several limitations to this study. Patient populations served by each specialty differ by varying amounts. Of note, HIV patients were prescribed TRT with greater frequency owing not to the prescribing physicians’ disregard of the ES guidelines but due to the unique needs of the patient population. Being a retrospective study, it was not feasible to examine the impact of emphasizing guidelines to clinicians or assess secondary outcomes resulting from TRT prescription outside of the recommendations of the ES guidelines. While patients with incomplete case data were excluded, oftentimes it was difficult to ascertain whether an instance of tests not being performed was a result of it not being ordered versus not being entered into the electronic medical record. Furthermore, not all laboratory tests were conducted at the same institution which introduces potential confounding from testing center variation. Lastly, the academic nature of the study facility limits the generalizability of the results to community-based practices. For example, the percentage of patients being treated by a male infertility specialist in this study is likely higher than for the average institution.

## Conclusion

The current study suggests that despite the presence of established guidelines for the management of patients with hypogonadism, adherence to ES recommendations may be low but varies by specialty. Urologists seem to follow guidelines most consistently, but greater emphasis to improve guideline-based management is needed among physicians treating hypogonadism. Future studies are needed to further confirm and understand discrepancies in practice patterns among providers, though specialty-specific guidelines are likely warranted.
